# Supplementary material for: Analysis of PPARγ Signaling Activity in Psoriasis
Source: Int J Mol Sci. 2021 Aug 10;22(16):8603. doi: 10.3390/ijms22168603 (PMC8395241; doi:10.3390/ijms22168603)
Supplement: Supplementary file 1 [file ijms-22-08603-s001.zip › Supplemental materials_Analysis of PPARg signaling activity in psoriasis/Pathway models/Models images and html files/Model of PPARG related pathways in psoriasis (short version)/4362.html]

fibrinogen


# Complex fibrinogen

|  |  |
| --- | --- |
| URN | urn:agi-gocomplex:0005577 |
| Total Entities | 3 |
| Connectivity | 4696 |
| Name | fibrinogen |
| ObjectType | Complex |

---

|  |  |
| --- | --- |
| ChildConcepts | FGA |
|  | FGB |
|  | FGG |

---

|  |  |
| --- | --- |
| Pathway | TLR4 Signaling in Leukocytes |
|  | Plasmin Effects in Inflammation |
|  | TAM Receptors in Platelet Aggregation |
|  | Platelet Activation via Adhesion Molecules |
|  | Scavenger Receptors in Platelet Aggregation |
|  | Platelet Activation via GPCR Signaling |
|  | Coagulation Cascade |
|  | Histidine-Rich Glycoprotein (HRG) |
|  | Positive Acute Phase Proteins Synthesis |
|  | Vascular Endothelial Cell Activation by Blood Coagulation Factors |
|  | Proteins Involved in Pulmonary Hypertension |
|  | Apoptotic Keratinocytes Clearance Recession in Systemic Lupus Erythematosus |
|  | Dendritic Cell Activation in Systemic Lupus Erythematosis |
|  | Neutrophil and Macrophage Function in Systemic Lupus Erythematosus |
|  | Macrophage Function in Diabetes Mellitus Type 1 |
|  | Antiphospholipid Antibodies in Platelets and Coagulation Cascade |
|  | Platelet and Coagulation Cascade Activation by Antiphospholipid Antibodies |
|  | Hereditary Thrombophilia and Pregnancy Complications |
|  | Proteins Involved in Thrombophilia |
|  | Thrombophilia Hereditary |
|  | SERPINA1 Associated Pulmonary Emphysema |
|  | Coagulation Factors and PAR1/2 Receptors (F2R/F2RL1) in Endometriosis |
|  | Proteins Involved in Chronic Obstructive Pulmonary Disease |
|  | Plasmin Role in Glomerulonephritis |
|  | Endothelial Cell Dysfunction in Glomerulonephritis |
|  | Integrins in Cancer Cell Motility, Invasion and Survival |
|  | MacrophageR -> CEBPB -> NF-kB Signaling |
|  | Fibrinogen Expression Targets |
|  | New Pathway (2) |
|  | Neighbors of fibrinogen |
|  | Model of PPARG signaling in psoriasis |
|  | PPARG negative regulators and targets |
|  | Model of PPARG related pathways in psoriasis (short version) |
|  | non-proteinal partners of SARSs |
|  | Dendritic Cell Activation in COVID19 |
|  | 1\_2\_Feedback relations between coagulation and fibrinolysis |
|  | 1\_1\_Dysfunction of coagulation cascade in hemophilia |
|  | 1\_SERPINA1-associated pulmonary emphysema |
|  | drug-target pairs direct regulation |

---

|  |  |
| --- | --- |
| Group | non protein partners |

---

|  |  |
| --- | --- |
| MedScan ID | 3105577 |

---

|  |  |
| --- | --- |
| Alias | clotting factor I |
|  | fibclot |
|  | blood clotting factor I |
|  | clotting factor 1 |
|  | fibrinogen |
|  | fibrinogen complexes |
|  | fibrinogen complex |
|  | clottagen |
|  | fibrin(ogen) |
|  | human fibrinogen |
|  | human fibrinogens |
|  | fibrinogens |
|  | blood clotting factor 1 |

---

|  |  |
| --- | --- |
| GO ID | 0005577 |

---
